# Perimyocarditis following first dose of the mRNA-1273 SARS-CoV-2 (Moderna) vaccine in a healthy young male: a case report

**DOI:** 10.1186/s12872-021-02183-3

**Published:** 2021-08-04

**Authors:** Ammar A. Hasnie, Usman A. Hasnie, Nirav Patel, Muhammad U. Aziz, Min Xie, Steven G. Lloyd, Sumanth D. Prabhu

**Affiliations:** 1 Department of Internal Medicine, University of Alabama at Birmingham (UAB), 1720 2nd Avenue S, BDB 327, Birmingham, AL 35233 USA; 2 Division of Cardiovascular Disease, Department of Medicine, University of Alabama at Birmingham, Birmingham, AL USA; 3 Department of Radiology, University of Alabama at Birmingham, Birmingham, AL USA; 4 Section of Cardiology, Birmingham Veterans Affairs Medical Center, Birmingham, AL USA

**Keywords:** COVID-19, Myopericarditis, Cardiac MRI, ECG, Case report

## Abstract

**Background:**

Half of U.S. adults have received at least one dose of the COVID-19 vaccines produced by either Pfizer, Moderna, or Johnson and Johnson, which represents a major milestone in the ongoing pandemic. Given the emergency use authorizations for these vaccines, their side effects and safety were assessed over a compressed time period. Hence, ongoing monitoring for vaccine-related adverse events is imperative for a full understanding and delineation of their safety profile.

**Case presentation:**

An 22-year-old Caucasian male presented to our hospital center complaining of pleuritic chest pain. Six months prior he had a mild case of COVID-19, but was otherwise healthy. He had received his first dose of the Moderna vaccine three days prior to developing symptoms. Laboratory analysis revealed a markedly elevated troponin and multiple imaging modalities during his hospitalization found evidence of wall motion abnormalities consistent with a diagnosis of perimyocarditis. He was started on aspirin and colchicine with marked improvement of his symptoms prior to discharge.

**Conclusions:**

We present a case of perimyocarditis that was temporally related to COVID-19 mRNA vaccination in an young male with prior COVID-19 infection but otherwise healthy. Our case report highlights an albeit rare but important adverse event for clinicians to be aware of. It also suggests a possible mechanism for the development of myocardial injury in our patient.

**Supplementary Information:**

The online version contains supplementary material available at 10.1186/s12872-021-02183-3.

## Background

When patients develop concomitant myocardial and pericardial injury, the clinical presentation can vary widely. The diagnosis of acute pericarditis requires at least two out of four diagnostic criteria including pericardial chest pain, pericardial rub, pericardial effusion, and diffuse ST-segment elevation or PR segment depression on electrocardiogram (ECG). Myocardial involvement can include elevated troponin levels, new ventricular dysfunction, or tissue abnormalities visualized on cardiac magnetic resonance imaging (CMR) [1]. When the primary symptoms are related to pericardial inflammation, this is referred to as myopericarditis. Meanwhile, patients can develop a perimyocarditis when significant myocardial injury is appreciated, including regional wall motion abnormalities along with pericardial symptoms as seen in our patient. This manuscript discusses a unique case of a patient who developed perimyocarditis that was temporally related to his first dose of the mRNA 1273 Sars-COV-2 vaccine.

## Case presentation

A 22-year-old male patient presented to our medical center with sharp substernal non-radiating chest pain for two days. He initially experienced generalized body aches and a subjective fever shortly after receiving his first dose of the mRNA-1273 (Moderna) vaccine three days prior. He then developed chest pain, at times pleuritic, which continued to worsen, prompting his presentation to the emergency department.

The patient had a mild COVID-19 infection six months prior to presentation. He took no medications or over-the-counter supplements. He denied any significant family history of chronic conditions including cardiovascular disease. He denied any tobacco, alcohol, or recreational drug use.

On initial presentation, his blood pressure was 97/57 mm Hg, heart rate 100 beats/min, respiratory rate 16 breaths/min, and oxygen saturation was 99 % on room air. He was afebrile with a temperature of 98.6° farenheight (38.6**°** celcius). On physical examination, he was tachycardic with a regular rhythm. No murmurs, rubs, or gallops were appreciated on auscultation. No jugular venous distension, tenderness to palpation of the chest wall, or lower extremity edema were noted.

Because of his pleuritic chest pain shortly after vaccination, our leading diagnosis was acute pericarditis. However, we also kept life-threatening causes such as acute coronary syndrome in our differential diagnosis.

**Fig. 1:**
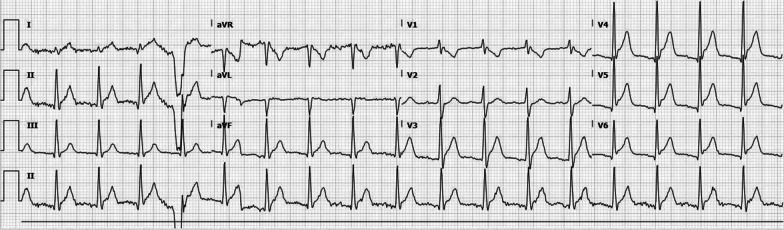
Initial ECG on admission showing diffuse ST elevations seen in leads V3–V6, I, II, and aVF. PR segment depression is noted in leads V3–V6

His ECG revealed normal sinus rhythm with diffuse ST elevation (Fig. 1). PR segment depression was discernible in leads V3–V6. High sensitivity troponin was 13,702 ng/L (normal range: 3–20 ng/L). B-type natriuretic peptide level was 48.0 pg/mL (normal range: < 100 pg/mL). His white blood cell count was 8700/cm^3^ with a neutrophilic predominance of 83%. A complete metabolic panel was unremarkable. The chest radiograph was unremarkable with no signs of focal consolidation, pneumothorax, or pleural effusion. A viral respiratory panel, HIV screen, and urine drug screen were all negative. Sars-CoV-2 nasopharyngeal polymerase chain reaction (PCR) testing was negative.

An echocardiogram revealed low-normal left ventricular ejection fraction (LVEF) of 50–55% with mid to apical anterior and anterolateral segments appearing hypokinetic (Additional file 1: Video 1).

**Fig. 2:**
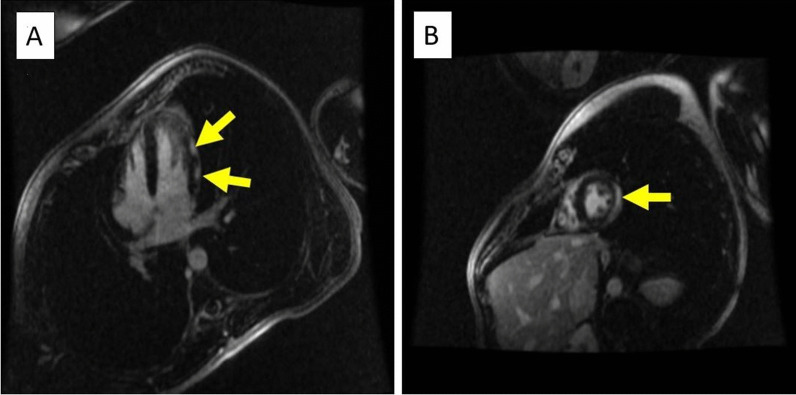
**a**, **b** Inversion recovery sequence in 4 chamber and short axis views 8 min following 0.2 mmol/kg IV of gadolinium contrast showing LGE in the subepicardial region of lateral LV wall. Arrows indicate areas of LGE

Left heart catheterization revealed angiographically normal coronary arteries. Left ventricular end-diastolic pressure was 18 mmHg. CMR showed a normal LVEF (58 %) with findings of dyssnchrony of the septal wall (Additional file 2: Video 2). It also showed subepicardial late gadolinium enhancement (LGE) involving the lateral wall and inferior segments at the midventricular and apical LV. Mild adjacent pericardial LGE was also noted (Fig. 2a, b). These findings were consistent with perimyocarditis.

**Fig. 3:**
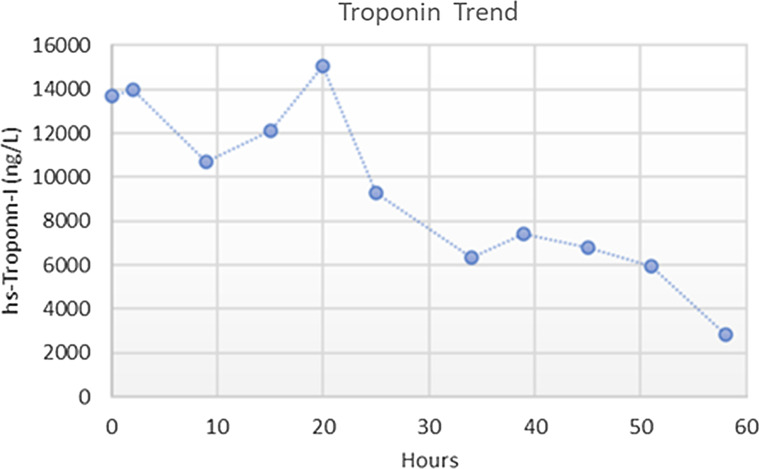
Graph showing high sensitivity troponin levels downtrending during the patient’s hospitalization

In the setting of pleuritic chest pain and anatomic pericardial involvement, the patient was treated with aspirin 650 mg three times per day and colchicine 0.6 mg twice per day. The patient was started on low dose metoprolol for myocardial protection after wall motion abnormalities were verified on CMR. His heart rate improved to a range of 70–80 beats per minute, troponin downtrended (Fig. 3), and there was complete resolution of his symptoms during the hospital stay.

Given the development of perimyocarditis after the first dose of the Moderna COVID-19 vaccine, we discussed the risks and benefits of receiving the 2nd dose of the vaccine. After careful consideration the patient elected to forego the 2nd dose of the vaccine. He was discharged with plans to complete a two week course of aspirin, three months of colchicine, and metoprolol. He was instructed to avoid alcohol intake and significant physical exertion for three to six months and to obtain cardiac clearance prior to returning to strenuous physical activity.

**Fig. 4:**
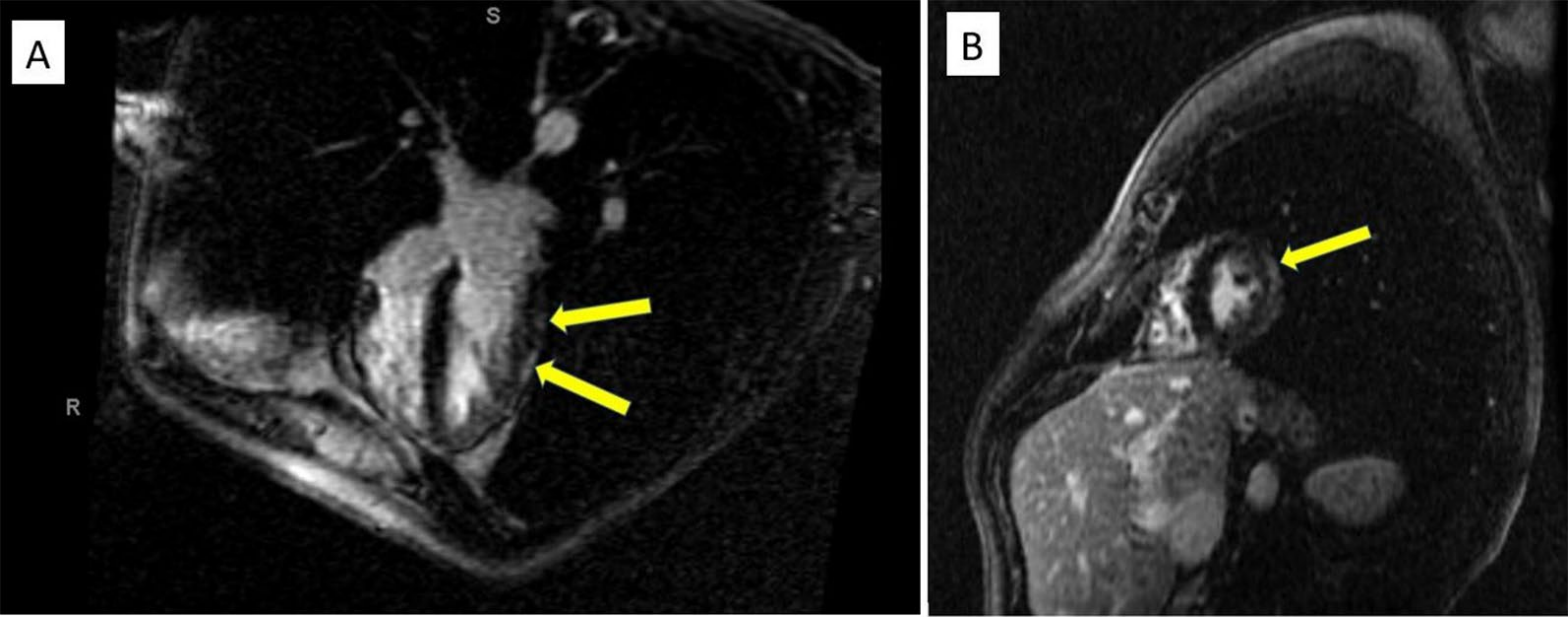
**a**, **b** The subepicardial delayed enhancement in the mid and distal segment of the left ventricular myocardium and adjacent pericardium have overall improved in size as well as intensity

He was seen one month following discharge with continued resolution of his symptoms. A repeat cardiac MRI was performed which showed improved subepicardial and adjacent pericardial delayed enhancement in the mid and distal left ventricle (Fig. 4a, b). It also showed resolution of the previously noted dyssnchrony suggestive of improving perimyocarditis.

## Discussion

The etiology of perimyocarditis can be categorized as infectious or non-infectious. In the developed world, viral infections remain the most common cause for developing both myocarditis and pericarditis [1]. The occurrence of perimyocarditis from a viral infection is secondary to direct cytotoxicity from the virus, impacting both the pericardium and myocardium. Within the subcategory of non-infectious causes, medications, immune-mediated diseases, and vaccine-associated myocarditis have been reported [1, 2].

Vaccine-associated myopericarditis involves signs and symptoms of myopericarditis within 30 days of a vaccine in the absence of other pathology [2]. Most cases of vaccine-associated myopericarditis have been reported following smallpox vaccination; however, isolated case reports have linked myopericarditis to the Tdap vaccine, varicella vaccine, and the influenza vaccine [3, 4].

Our patient developed acute perimyocarditis three days after receiving his first dose of the Moderna vaccine. The emergency use authorization for the Moderna vaccine was issued in December, 2020. Clinical trials have demonstrated 94.1% efficacy of the vaccine in preventing COVID-19 illness, including severe disease with a strong safety profile [5].

The mRNA-1273 vaccine is composed of a modified mRNA encoding the spike protein of SARS-CoV-2, and when incorporated, directs the cell to synthesize spike protein. Immune cells then detect the COVID spikes and develop protective antibodies to prevent viral replication [5]. While this represents a newer vaccine approach, mRNA vaccines do not cause COVID-19 as the mRNA breaks down quickly in the cell and the vaccine encodes only a part of the complete virion. However, in the absence of a prior large-scale mRNA vaccine utilization, adverse events are difficult to predict. Isolated cases have been reported which described predominately young, healthy males who developed acute myocarditis following COVID-19 mRNA vaccination. Patients typically developed symptoms within days of receiving the second dose of their vaccine or, as seen in our case, the first dose in patients who were previously infected with COVID-19 [6–8]. Fortunately, the disease course of these patients was mild and recovered without major complications.

Our patient denied any hypersensitivity reactions to previous vaccines. However, his immune system may have been primed from his earlier SARS-CoV-2 exposure during mild COVID-19 disease, which may have precipitated his clinical presentation. An ongoing clinical study comparing reactogenicity between patients previously infected with SARS-CoV-2 versus naïve individuals found that the former experienced systemic side effects (i.e., fatigue, headache, and chills) with higher frequency. SARS-CoV-2 spike IgG titers before and after first dose of an mRNA vaccine (either Moderna or Pfizer) was also studied. Sampling found that patients with previous COVID-19 disease had antibody titers 10 to 45 times higher compared to those without previous infection [9]. The ZOE Covid Symptom Study, a smartphone application, also has a dataset where participants log their symptoms from COVID-19 itself to symptoms post-vaccination. They found participants previously infected with COVID-19 experienced more systemic adverse effects than naïve participants (33 % vs. 19 %) [10].

The pathophysiology of vaccine-associated myocarditis is not well defined. However, in COVID-19 myocardial inflammation, SARS-CoV-2 utilizes the spike protein to bind to a membrane-bound form of angiotensin-converting enzyme 2 (ACE2), facilitating intracellular uptake. Cardiovascular tissues express a large proportion of ACE2 receptors and therefore become a target for viral replication and direct viral injury [11, 12]. Naive T lymphocytes can be subsequently primed through viral antigens, and potentially self proteins released from damaged cardiomyocytes, via antigen-presenting cells. Uncommonly, this can lead to primed T-lymphocyte migration to cardiovascular tissues, cell-mediated cytotoxicity, and lymphocytic myocarditis [13]. Pro-inflammatory cytokines are then released which augments T-lymphocyte activation and furthers damage [11]. We propose that our patient’s initial SARS-CoV-2 infection primed T-lymphocytes against both SARS-CoV-2 proteins and cardiac antigens released during sub-clinical myocardial inflammation during his initial infection. His first dose of the Moderna vaccine may have reactivated this intrinsic immune response, subsequently resulting in myocardial injury. It’s also worth noting early serologic data found patients with prior natural infection developed higher antibody titers following first dose of an mRNA vaccine. This could represent an alternative vaccine related immune mechanism which could predispose patients with prior COVID-19 infection to a higher incidence of myocarditis compared to naïve patients. Obviously, the mechanism of COVID-19 vaccine associated myocarditis will require further study for conclusive definition.

As our patient remained clinically stable, he did not require endomyocardial biopsy during his hospitalization. He was treated with aspirin and colchicine as anti-inflammatory therapy remains the cornerstone of treatment for acute pericarditis [14]. His imaging showed no signs of ventricular dysfunction so he was not started on guideline-directed medical therapy for heart failure. In the setting of his acute myocarditis we elected to start metoprolol to help prevent heart failure, life-threatening arrythmias, and also limit ‘mechanical inflammation’ [15]. In addition, we recommended abstinence from strenuous activity to decrease risk of remodeling and sudden death as well [16]. While the benefits of vaccination are substantial, given the hypersensitivity myocarditis he suffered following the first dose of the Moderna vaccine and lack of information available at the time, after a patient-centered discussion he elected to forego the second dose of the vaccine.

## Conclusions

This case highlights a rare adverse event of perimyocarditis with the Moderna vaccine against SARS-CoV-2. While our knowledge of COVID-19 mRNA vaccine-related cardiovascular injury is limited, clinicians should be aware of this possibility. A registry of cardiovascular-related complications will be of benefit to define the incidence of SARS-CoV-2 vaccine-associated cardiovascular adverse events. This case raises an important question on further management for patients who experienced acute myocarditis following the first dose of the COVID-19 vaccination in regard to subsequent booster vaccinations and requires further investigation.

## Supplementary Information

**Additional file 1: Video 1**. Initial echocardiogram showing mild hypokinesis of the mid to apical anterior and anterolateral segments.

**Additional file 2: Video 2**. Cardiac MRI completed on hospital day 2 showing areas of hypokinesis and dssynchrony in distal septum and of the RV. Sequence details: SSFP technique #phase 20; flip angle 45°, slice thickness 8 mm, FOV 40 cm, TR-3.7 ms, TE-1.5 ms.

## Data Availability

Not applicable.

## Abbreviations

ECG: Electrocardiogram
Cardiac MRI or CMR: Cardiac Magnetic Resonance Imaging
PCR: Polymerase Chain Reaction
LVEF: Left Ventricular Ejection Fraction
LGE: Late Gadolinium Enhancement
ACE2: Angiotensin-Converting Enzyme 2
